# Western diet feeding influences gut microbiota profiles in apoE knockout mice

**DOI:** 10.1186/s12944-018-0811-8

**Published:** 2018-07-18

**Authors:** Baoning Liu, Yali Zhang, Rong Wang, Yingfeng An, Weiman Gao, Liang Bai, Yandong Li, Sihai Zhao, Jianglin Fan, Enqi Liu

**Affiliations:** 1 Laboratory Animal Center, Xi’an Jiaotong University Health Science Center, Xi’an, 710061 Shaanxi China; 2 Research Institute of Atherosclerotic Disease, Xi’an Jiaotong University Cardiovascular Research Center, Xi’an, 710061 Shaanxi China; 3 Shaanxi Province Centre for Disease Control and Prevention, Xi’an, 710054 Shaanxi China; 4 Department of Pathology, First Affiliated Hospital of Xi’an Medical University, Xi’an, 710000 Shaanxi China; 5 Department of Molecular Pathology, Faculty of Medicine, Interdisciplinary Graduate School of Medicine, University of Yamanashi, Yamanashi, 409-3898 Japan

**Keywords:** Gut microbiota, apoE KO mice, Atherosclerosis, Western diet

## Abstract

**Background:**

Gut microbiota plays an important role in many metabolic diseases such as diabetes and atherosclerosis. Apolipoprotein E (apoE) knock-out (KO) mice are frequently used for the study of hyperlipidemia and atherosclerosis. However, it is unknown whether apoE KO mice have altered gut microbiota when challenged with a Western diet.

**Methods:**

In the current study, we assessed the gut microbiota profiling of apoE KO mice and compared with wild-type mice fed either a normal chow or Western diet for 12 weeks using 16S pyrosequencing.

**Results:**

On a western diet, the gut microbiota diversity was significantly decreased in apoE KO mice compared with wild type (WT) mice. *Firmicutes* and *Erysipelotrichaceae* were significantly increased in WT mice but *Erysipelotrichaceae* was unchanged in apoE KO mice on a Western diet. The weighted UniFrac principal coordinate analysis exhibited clear separation between WT and apoE KO mice on the first vector (58.6%) with significant changes of two dominant phyla (*Bacteroidetes* and *Firmicutes*) and seven dominant families (*Porphyromonadaceae*, *Lachnospiraceae, Ruminococcaceae, Desulfovibrionaceae, Helicobacteraceae, Erysipelotrichaceae* and *Veillonellaceae*). *Lachnospiraceae* was significantly enriched in apoE KO mice on a Western diet. In addition, *Lachnospiraceae* and *Ruminococcaceae* were positively correlated with relative atherosclerosis lesion size in apoE KO.

**Conclusions:**

Collectively, our study showed that there are marked changes in the gut microbiota of apoE KO mice, particularly challenged with a Western diet and these alterations may be possibly associated with atherosclerosis.

**Electronic supplementary material:**

The online version of this article (10.1186/s12944-018-0811-8) contains supplementary material, which is available to authorized users.

## Background

Excess intake of a high-fat diet or Western diet is generally considered unhealthy because such a diet can cause many metabolic disorders such as obesity, diabetes and atherosclerosis [1–3]. Although this notion has been widely accepted, the underlying mechanisms responsible for these pathophysiological consequences are still not fully understood. Gut microbiota has received more attention for its roles in both physiological and pathophysiological processes [4–7]. It has been shown that many metabolic diseases are related with the changes in gut microbiota. Laboratory mice are frequently used for the study of human diseases but most strains of wild-type mice do not show any metabolic disorders on a normal chow diet. However, if WT mice were fed with a diet containing 21% fat along with 0.15% cholesterol, which is often terminated as a Western diet, they show elevated plasma lipids but they do not show obvious lesions of atherosclerosis. However, if mice are genetically deficient in apolipoprotein E (apoE) such as apoE knock-out (KO) mice, they are sensitive to a Western diet and rapidly develop hyperlipidemia and atherosclerosis. ApoE is a component of all lipoproteins except for low density lipoproteins (LDL) and functions as a ligand for LDL receptors and LDL-related proteins (LRP) on the liver thereby playing an important role in the catabolism of the remnants of chylomicrons and very low-density lipoproteins. The genetic deficiency of apoE gene leads to the accumulation of cholesterol-rich remnants in plasma in both humans and animals [8–12]. Therefore, Western diet-fed apoE KO mice are widely used to study many facets of human hyperlipidemia and atherosclerosis [13–16].

Given the fact that the gut microbiota plays an important role in many metabolic diseases such as obesity, diabetes, metabolic syndrome [17–20] and atherosclerosis [21–24], we envisioned that the gut microbiota may be altered in apoE KO mice when challenged with a Western diet. Driven by the popularization of high throughput sequencing-based approaches, many meta-genomic studies were carried out to characterize the profile changes of the gut microbiome in atherosclerotic individuals [22, 23] and facilitated the comprehensive understanding of the interaction between the gut microbiota and host physiology. However, as the most commonly used atherogenic mouse model, the gut microbiota of apoE KO mice compared with WT mice has not been clarified and their relationship with metabolic and pathophysiological consequences remain unclear. In the current study, we investigated the gut microbiota profiling of apoE KO mice fed a chow and western diet compared with WT mice. Our data revealed that apoE KO mice exhibited unique features of the gut microbiota diversity and compositions and these features may provide novel insight into understanding the relationship between gut microbiota and atherosclerosis.

## Methods

### Animals

In this study, six-week-old male C57BL/6 J mice (wild type, WT) and apoE KO were used and divided into four groups: WT mice feed on normal chow diet (WT-CD), WT mice feed on western diet (WT-HC), apoE^−/−^ mice feed on normal chow diet (apoE-CD), and apoE KO mice feed on Western diet (apoE-HC) (*n* = 7~ 8). The composition of normal chow diet is based on the NIH feeds standard (NIH-07, NSN-8710-00-509-7915). The Western diet contains 21% fat and 0.15% cholesterol. The diets were provided by Beijing Keao Xieli Feed Co.,Ltd. All animals were provided ad libitum access to food and water and housed under the conventional 12 h: 12 h light/dark cycle for 12 weeks. The plasma total cholesterol (TC), Triglyceride (TG) and high-density lipoprotein cholesterol (HDL-C) levels were measured at 0, 4, 8, 12 weeks from the beginning of experiment. At the end of the experiment, all mice were sacrificed by a sodium pentobarbital overdose. The heart and artery tissue and intestinal contents were collected for further processing. Animal care and experimental procedures were approved by the Animal Care and Use Committee of Xi’an Jiaotong University.

### Plasma lipid measurement

Mice were fasted for 8 h before collecting the blood samples from tail veins. Then blood samples were centrifuged at 3000 g for 15 min and supernatants were collected. The plasma levels of TC, TG, and HDL-C were measured with commercially available kits from Biosino Bio-technology and Science (Beijing) according to the manufacturer’s protocols.

### Analysis of atherosclerotic lesions

Mice were euthanized by a sodium pentobarbital overdose and flushed with saline at physiological pressure, then perfused with 4% paraformaldehyde through the left ventricle. The heart and aorta were dissected, removed and fixed with parafonnaldehyde. Aortas were open longitudinally, stained with Oil Red O and digitally scanned. En face lesion area was measured with WinROOF 6.5 (Mitani Co., Fukui, Japan). To quantify atherosclerosis at the aortic root, cross-cryosections were prepared according to our previous report [25]. In brief, part of each heart between the lower tips of the right and left atria were embedded in optimal cutting temperature compound and the aortic root was sectioned (8 μm) serially. Corresponding sections on separate slides were treated with Oil Red O staining and analyzed by WinROOF 6.5.

### Immunohistochemistry analysis

For immunohistochemistry, corresponding sections were incubated with 3% hydrogen peroxide for 30 min and blocked with 2% horse serum for 2 h. Sections were then stained with anti-MOMA2 (Cat.No.ab-33,451, abcam) or anti-α-SMA (AA132, Beyotime Biotech Inc) at 4 °C for overnight. The sections were then incubated with a secondary antibody against rat IgG (Thermo Scientific) for 1 h at room temperature. The sections were visualized with a DAB substrate kit (Vector Laboratories). Finally, image processing was carried out using a Leica DMRE microscope equipped with Spot digital image analysis software and camera.

### Gut bacterial DNA extraction and sequencing

About each of 0.5 g cecal sample was used for DNA extraction with QIAamp Fast DNA Stool Mini kit (Qiagen) according to the manufacturer’s protocols. The extracted DNA was amplified with primers targeting V4-V5 hypervariable region of bacterial 16S rRNA genes (forward: 5-CCAGCAGCYGCGGTAAN-3; reverse: 5-CCGTCAATTCNTTTRAGT-3). PCR were then performed under the following condition: denaturation (95 °C, 3 mins), amplification (25 cycles), annealing (55 °C, 30 s), and elongation (72 °C, 30 s), and final elongation (72 °C, 5 mins). Sequencing was performed on the platform of Illumina HiSeq 2000. 16S rRNA sequence were explored by the package of Quantitative Insights Into Microbial Ecology (QIIME, 1.9.0) [26]. To avoid GC bias and low-quality nucleotides, the raw sequences were filtered with the following criteria: minimum Phred score, 20; minimum number of high-quality calls, 0.75; maximum number of consecutive low-quality base calls, 3. Trimmed sequences were then assembled [27].

### Gut microbiota diversity and composition analysis

Sequence with 97% similarity were clustered into one operational taxonomic unit by UCLUST [28]. The closed-reference taxonomy was assigned to genus level against the Greengenes database [29] with 0.5 confidence threshold. Sequence occurred less than three times were removed from further analysis. Estimation of Shannon-Weiner index based on 16S rRNA gene were performed to assess the gut microbiota diversity with an average sequence number of 2,4000 reads per sample. Weighted uniFrac distance [30, 31] were used for the principle coordinate analysis.

### Identification of gut microbiota features that discriminate groups

Linear Discriminant Analysis Effect Size (LEfSe) were performed to identify the bacterial communities that differentiate different groups of mice [32]. Gut bacterial richness was used to calculate the effect size on either dietary or apoE deficiency. The threshold on logarithmic Linear Discriminant Analysis (LDA) score were set as 2 (default). One-way ANOVA was used to analyze differences between groups and Tukey’s multiple comparison test was applied to correct for multiple tests.

### Statistics

The TG, TC and HLD-C levels were represented as mean ± SEM. The richness of dominant gut bacteria was presented by average and relative abundance with more than 0.5% were considered as dominate bacterial communities. Statistical analyses were performed by one-way ANOVA followed by Student’s t-test. *P* < 0.05 was considered as statistics significant. Pearson’s correlation was calculated for each pairwise combination of each gut bacterial family versus atherosclerosis lesion area ratio using Graphpad Prism 6.

## Results

### Dyslipidemia and atherosclerosis in apoE KO mice

**Fig. 1:**
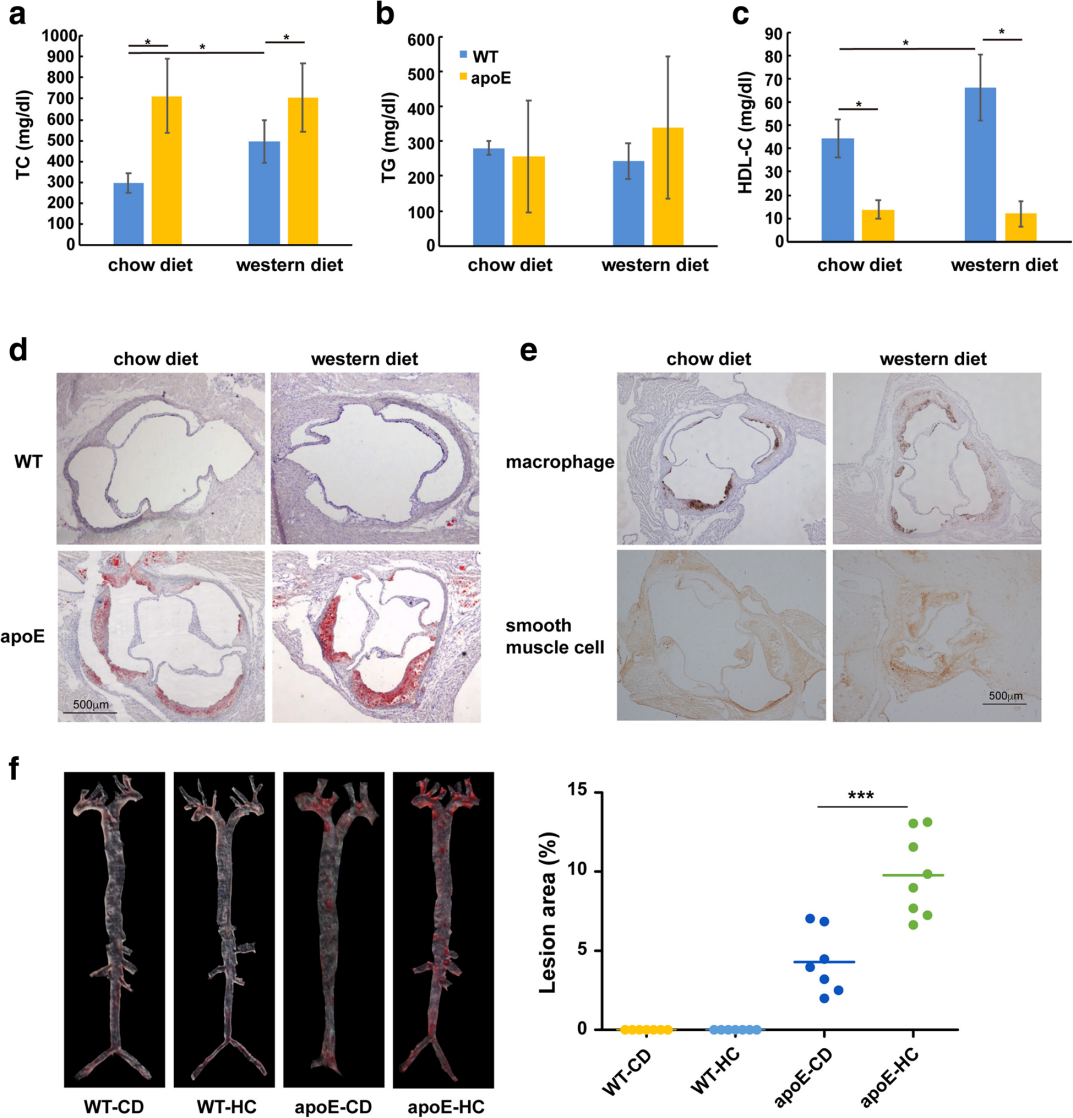
Western diet and apoE gene deletion elevate blood lipids and aggravate atherosclerosis. **a** Total cholesterol (TC); (**b**) Triglycerides (TG); (**c**) High-density lipoprotein cholesterol (HDL-C) levels at 12 weeks in the plasma samples from four groups of mice; (**d**) Cross sections of aortic roots from four groups of mice were stained with Oil-Red-O; (**e**) Macrophage and smooth muscle cells staining of apoE KO mice; (**f**) En face Oil-Red-O staining of aortic specimens with the quantitive analysis on the right panel. Data are expressed as the mean ± SEM, *n* = 7~ 8 for each group. ^∗^*P* < 005, ^∗∗∗^*P* < 0001 versus wild type (WT) mice

On a normal chow diet, apoE KO mice had significantly higher levels of plasma TC but lower levels of HDL-C than WT mice but TG levels were unchanged (Fig. 1a, b and c). On a Western diet, TC levels of WT mice were increased but no change in apoE KO mice compared to normal chow diet (Fig. 1a). The same trend was also found in HDL-C (Fig. 1c). In spite of this, there were no aortic lesions in WT mice on both a chow and Western diet but atherosclerosis was observed in apoE KO mice on both a chow and Western diet (Fig. 1d, e and f). Atherosclerotic lesions were significantly increased in Western diet-fed apoE KO mice compared with chow-fed apoE KO mice (Fig. 1d, e and f). Moreover, the macrophage and smooth muscle cells increased in high cholesterol feeding apoE KO mice compared to normal chow diet (Fig. 1e).

### The gut microbiota alteration in apoE KO mice

**Fig. 2:**
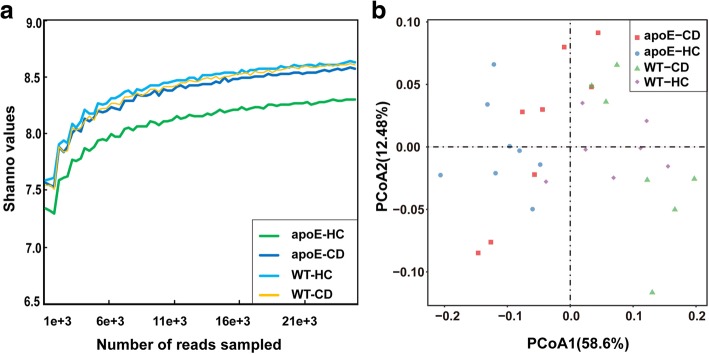
The gut microbiota diversity and PCoA analysis of four groups of mice. **a** The comparison of Shannon-Weiner index, representing the microbiota diversity for four groups of mice; (**b**) Principal coordinate analysis (PCoA) for gut microbiota of four groups of mice

**Fig. 3:**
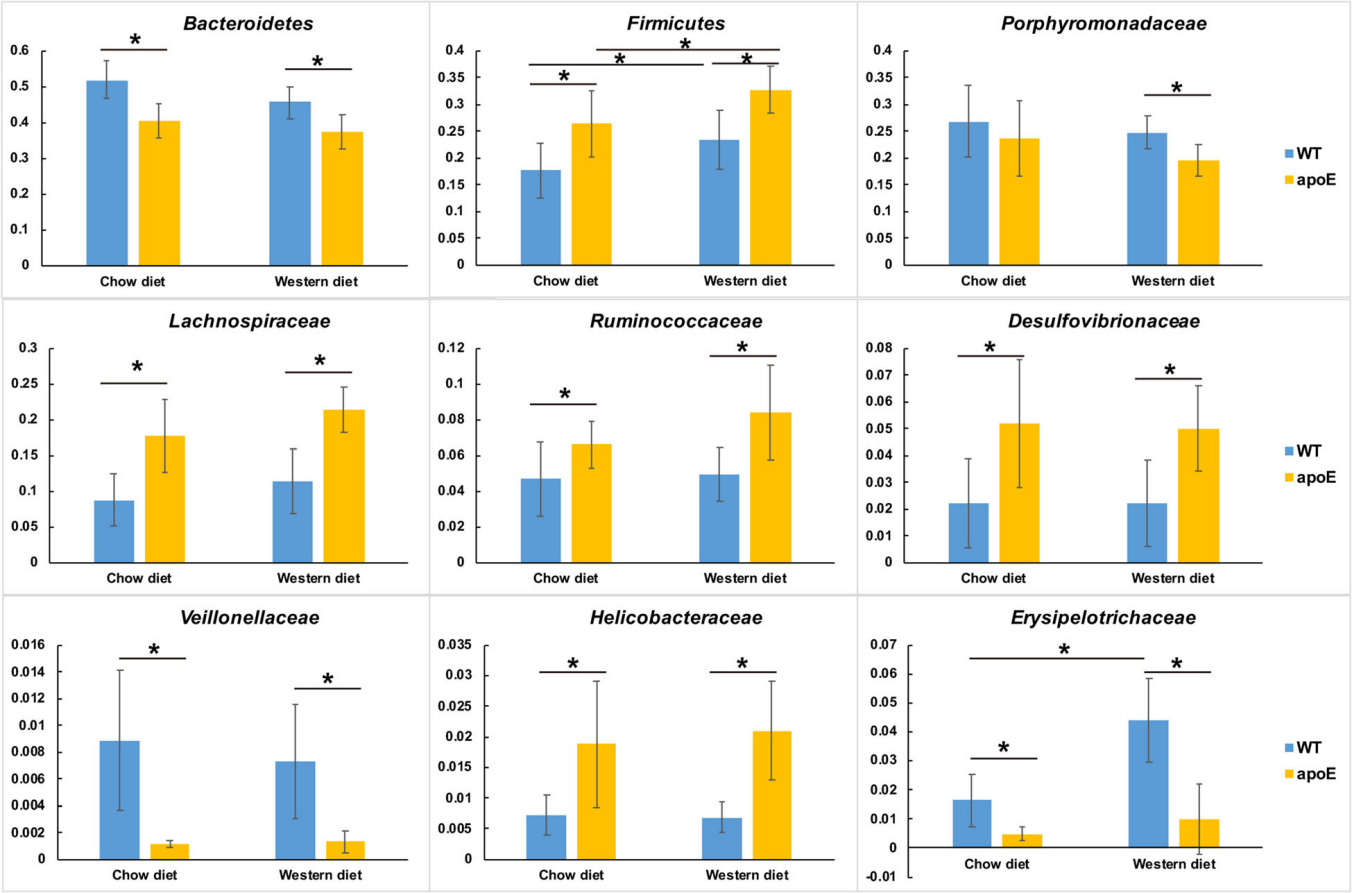
Comparison of gut microbiota among four groups of mice. *T*-test was applied to detect the statistical significance. Data are expressed as the mean ± SEM, *n* = 7~ 8 for each group. ^∗^*P* < 005 versus wild type (WT) mice

To shed light on the gut microbiota distribution in mice, we sequenced V4-V5 amplicons of 16S rRNA genes. After quality control, we obtained a total of 2,766,806 sequences (69,170 ± 26,490 per sample) with the average length of 327 bp (Additional file 1: Figure S1). As expected, phylum-level composition was dominated by three phyla (*Bacteroidetes*, *Firmicutes*, and *Proteobacteria*), accounting for 84.7%~ 87.1% of the gut bacterial community (Additional file 2: Figure S2). Of these phyla, the most dominant phylum was *Bacteroidetes* with a richness of 36.4%~ 50.9%, followed by *Firmicutes* with 17.6%~ 30.6%, and *Proteobacteria* with 15.6~ 20.1% (Additional file 2: Figure S2). The other phyla such as *Actinobacteria*, *Deferribacteres* and *Verrucomicrobia* also were classified, but these bacteria were present at relatively very low abundance (less than 0.5%). Family-level composition was dominated by *Lachnospiraceae, Porphyromonadaceae*, *Prevotellaceae*, *Ruminococcaceae*, *Geobacteraceae*, *Desulfovibrionaceae* and *Bacteroidaceae* (Additional file 3: Figure S3). Estimation of Shannon-Weiner index, a measurement of the α-diversity of the gut microbiota, demonstrated that apoE KO mice had a tendency for decreasing the microbiota diversity when challenged with a Western diet (Fig. 2a) whereas it was unchanged in WT mice fed on a Western diet. Principal coordinate analysis based on weighted-unifrac distance showed a high degree of divergence between apoE KO and WT mice on the first vector, which accounts for 58.6% of the gut microbiota profile (Fig. 2b) although the gut microbiota change induced by different diets was indistinguishable (Fig. 2b). On normal chow diet, apoE KO mice showed significant variance of two phyla (*Firmicutes* and *Bacteroidetes*) and six families (*Lachnospiraceae, Ruminococcaceae, Desulfovibrionaceae, Helicobacteraceae, Erysipelotrichaceae* and *Veillonellaceae*) (Fig. 3) compared to WT mice. Western diet feeding led to the increase of *Firmicutes* and *Erysipelotrichaceae* in WT mice but *Erysipelotrichaceae* was not changed in apoE KO mice (Fig. 3).

### LEfSe and correlation analysis

**Fig. 4:**
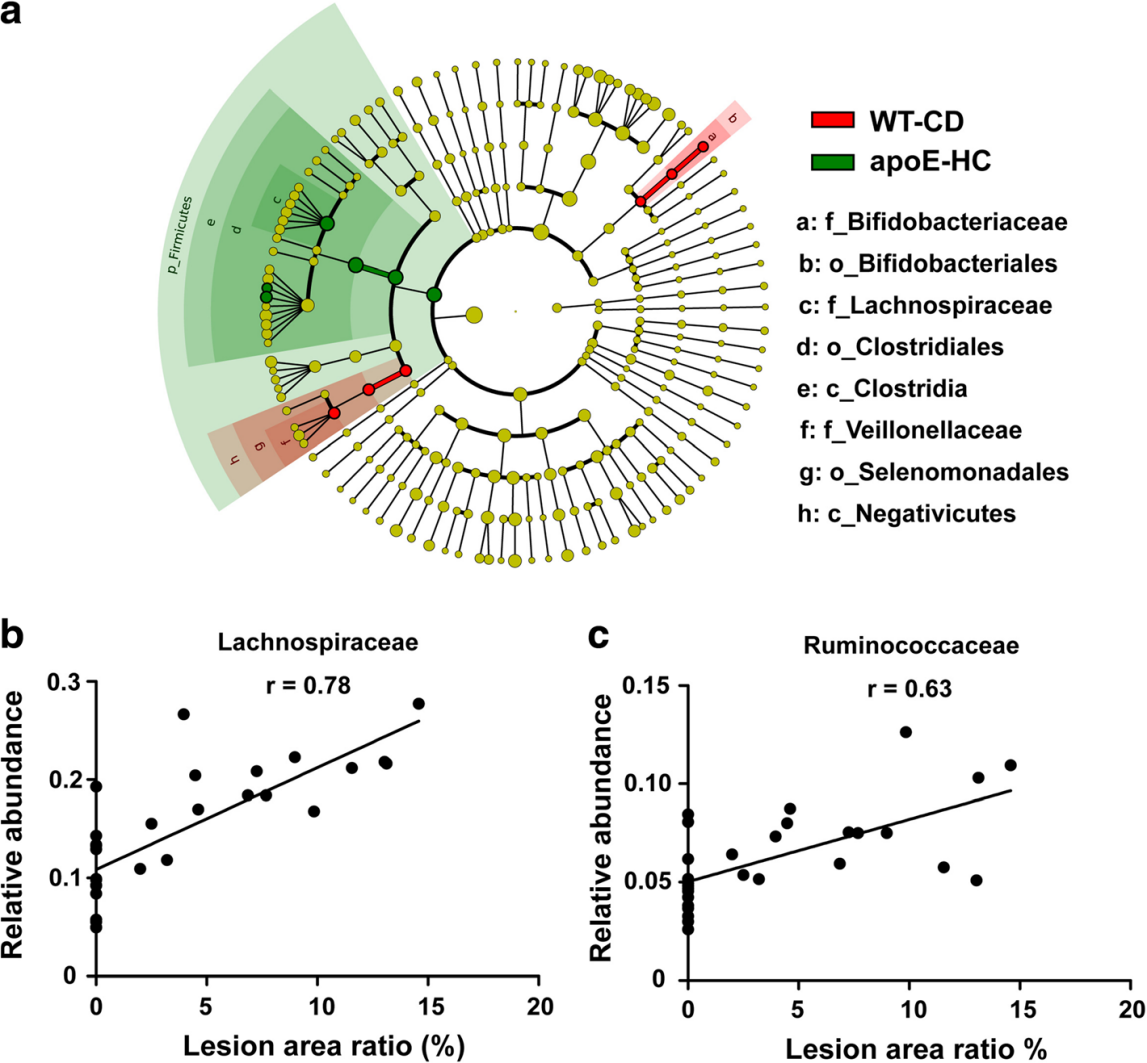
Linear regression plots and LDA Effect Size (LEfSe) analysis. **a** Cladogram generated with Linear Discriminant Analysis (LAD) Effect Size (LEfSe) analysis illustrating significant shifts in the gut microbiota in the four groups of mice (n = 7~ 8). Size of yellow circles is proportionated to each taxon’s mean relative abundance; (**b**) Linear regression of *Lachnospiracea* correlated with relative atherosclerosis lesions; (**c**) Linear regression of *Ruminococcaceae* correlated with relative atherosclerosis lesions. *P* < 0.05 and Pearson’s correlation coefficient (r) > 0.6

The strict version of LEfSe (all against all) detected eight gut bacterial clades showing significant differences between aopE KO mice fed a Western diet and WT mice on a normal chow diet with LDA scores higher than two (Fig. 4a). The phylum of *Firmicutes* and *Lachnospiraceae* (from class to family) were found to be enriched in apoE KO mice on a Western diet, while *Bifidobacteriales* (from order to family), *Negativicutes*, *Selenomonadales* and *Veillonellaceae* were enriched in WT mice on a normal chow diet (Fig. 4a). In order to explore whether gut microbiota have any correlations with atherosclerosis in apoE KO mice, we performed a correlation analysis of family-level gut microbiota and aortic atherosclerosis lesion area. The relative abundances of *Lachnospiraceae* and *Ruminococcaceae* were found to be positively correlated with atherosclerosis lesion area (*P* < 0.05) (Fig. 4b and c).

## Discussion

In the current study, we analyzed the gut microbiota of apoE KO mice and compared with WT mice on either a normal chow or Western diet. It is well known that apoE KO mice can develop atherosclerosis even on a normal chow diet and moreover, a Western diet is very atherogenic and accelerates the development of atherosclerosis in apoE KO mice. In spite of this, it is not clear whether Western diet feeding influences the gut microbiota in apoE KO mice or whether there are any associations between the altered gut microbiota profiling with atherosclerosis. Our results showed that the gut microbiota diversity and composition were remarkably changed in apoE KO mice, especially on a Western diet. *Firmicutes* and *Clostridia* (from class to family) were found to be enriched in apoE KO mice on a Western diet by LEfSe analysis. It has been reported that the relative abundance of *Firmicutes* could lead to the formation of increased amounts of metabolic endotoxins like lipopolysaccharides, which was able to enter the blood steam and cause the chronic inflammation [33]. Three species, including *Clostridium asparagiforme*, *Clostridium hathewayi*, and *Clostridium sporogenes* in the family of *Clostridiales*, were also reported to be correlated with the accumulation of trimethylamine N-oxide (TMAO) [34, 35]. Atherosclerosis is considered as a chronic inflammatory process and previous studies have identified that the gut microbiota-dependent TMAO production directly contributed to the progression of atherosclerosis, indicating that gut microbiota participates in the development of atherosclerosis [24, 35–37]. TMAO was able to accelerate atherosclerosis by disrupting the cholesterol transport and modifying bile acids composition [38], enhancing thrombosis formation by activating platelets [39], and increasing high blood pressure induced heart failure by adverse cardiac remodeling [40]. In our study, *Lachnospiraceae* and *Ruminococcaceae*, the two most abundant families in the order of *Clostridiales*, were found to be correlated with the atherosclerosis size. Although it is not possible to ascertain whether such correlation is causal or casual, the further studies are required to investigate its significance in future. Nevertheless, it has also been reported that the members of *Lachnospiraceae* were able to cause diabetes mellitus in germ-free mice [41]. Therefore, the gut microbiota transplantation would be performed using germ-free mice to determine the causal relationship in the future.

## Conclusions

Collectively, our study showed that there are marked changes in the gut microbiota of apoE KO mice, particularly challenged with a Western diet and these alterations may be possibly associated with atherosclerosis.

## Additional files

Additional file 1: **Figure S1.** Reads number distribution of each sample based on 16S rRNA gene sequencing. (JPG 262 kb)

Additional file 2: **Figure S2.** Dominant gut microbiota compositions of samples at phyla level. (JPG 96 kb)

Additional file 3: **Figure S3.** Dominant gut microbiota compositions of samples at family level. (JPG 144 kb)
