# Supplementary figures and images for: Western diet feeding influences gut microbiota profiles in apoE knockout mice
Source: Lipids Health Dis. 2018 Jul 18;17:159. doi: 10.1186/s12944-018-0811-8 (PMC6052692; doi:10.1186/s12944-018-0811-8)

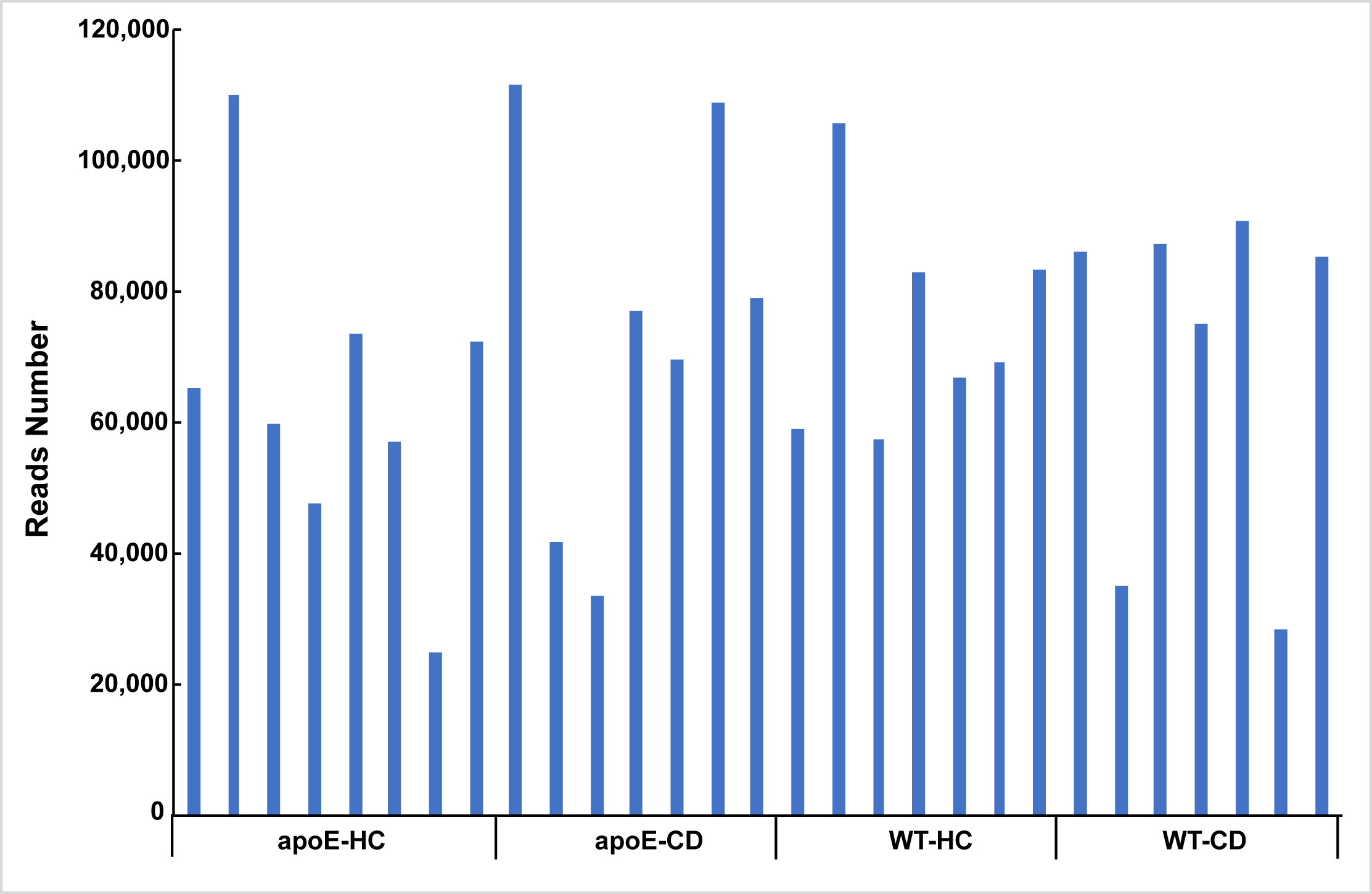

Supplement: Supplementary file 1 — Figure S1. Reads number distribution of each sample based on 16S rRNA gene sequencing. (JPG 262 kb) [file 12944_2018_811_MOESM1_ESM.jpg]

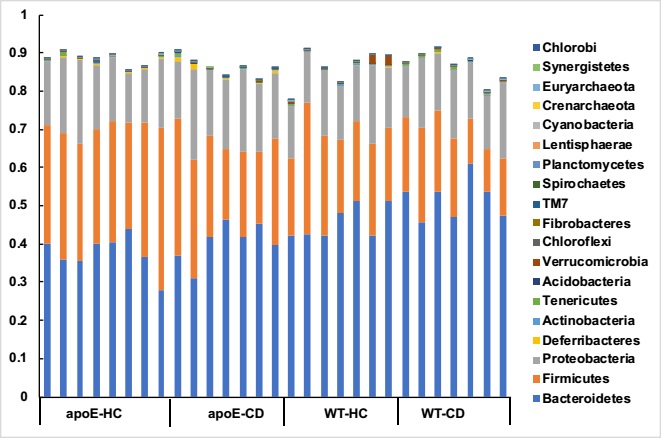

Supplement: Supplementary file 2 — Figure S2. Dominant gut microbiota compositions of samples at phyla level. (JPG 96 kb) [file 12944_2018_811_MOESM2_ESM.jpg]

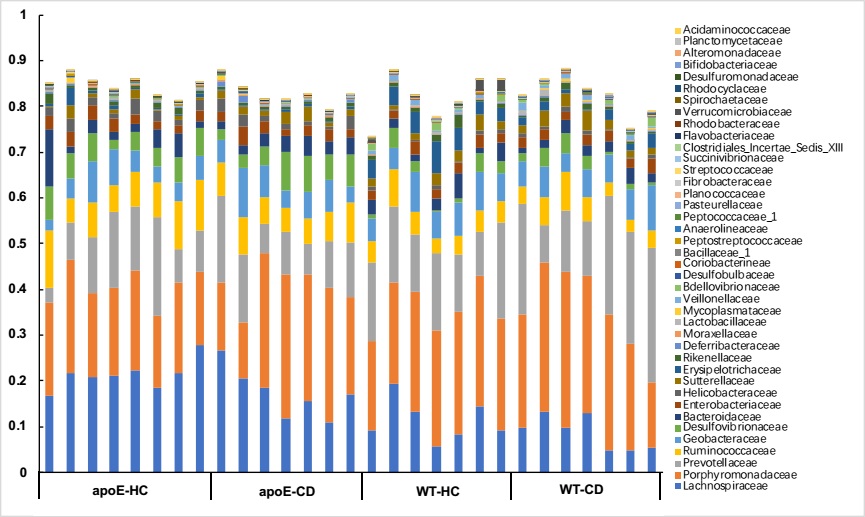

Supplement: Supplementary file 3 — Figure S3. Dominant gut microbiota compositions of samples at family level. (JPG 144 kb) [file 12944_2018_811_MOESM3_ESM.jpg]
